# Spatiotemporal aggregation and population distribution characteristics of HIV/AIDS in Nanchang city: A monitoring analysis from 2012–2021

**DOI:** 10.1371/journal.pone.0342375

**Published:** 2026-02-05

**Authors:** Li Wang, Liang Lu, Zhibin Tu, Liping Qiu, Xiaoqin Tong, Jinyi Sun, Rui Liu

**Affiliations:** 1 Nanchang Center for Disease Control and Prevention, Nanchang, China; 2 Shenzhen Bao’an District Chronic Disease Prevention and Treatment Hospital, Shenzhen, China; Xiangya Hospital Central South University, CHINA

## Abstract

AIDS is a major public health issue. As a transportation and economic hub in central China, Nanchang has frequent population mobility and unbalanced regional development, making it urgent to explore the prevalence characteristics of HIV/AIDS. Based on HIV/AIDS surveillance data in Nanchang from 2012 to 2021, this study innovatively integrates the Joinpoint regression model with spatiotemporal geographic information technology to construct an epidemiological analysis framework of “time-space-population”. The results show that a total of 3,789 HIV/AIDS infection cases were reported in the decade, with the growth rate of new cases (5.4%) significantly higher than the population growth rate (2.1%). There is a negative correlation between incidence and mortality, and men and the elderly (≥65 years old) are at prominent infection risk. Spatially, cases show significant heterogeneity, with “high-high” clustering areas concentrated in the city center and gradually expanding to surrounding areas, which is related to factors such as population density. The study provides a scientific basis for targeted interventions for men and the elderly, optimization of prevention and control strategies, and regional collaboration.

## Introduction

Acquired immunodeficiency syndrome (AIDS), an immunodeficiency disease caused by human immunodeficiency virus (HIV) infection, is characterized by a high fatality rate and long-term harm and has become one of the most serious public health and social problems [1,2]. The disease burden of HIV/AIDS remains high. The most common treatment regimens, such as antiretroviral therapy (ART), can only effectively and durably suppress the replication of HIV and improve the survival rate, but they cannot eliminate the virus or achieve a clinical cure [3,4]. Prevention is widely recognized as the most cost-effective strategy to curb the epidemic’s spread, as it directly reduces new infections, eases the burden on healthcare systems, and protects vulnerable populations from exposure risks. However, existing prevention efforts often lack spatial targeting: they tend to adopt generalized, one-size-fits-all approaches such as universal public health campaigns rather than tailoring interventions to the specific geographic contexts where infections cluster. Some studies have indicated that geographic disparities and social factors together contribute to the spatial and temporal heterogeneity in the spread and transmission of HIV/AIDS [5,6]. However, the analysis of the spatial attributes of HIV/AIDS in small and medium-sized cities (SMSC) remains insufficient in depth, and an integrated “time-space-population” analytical framework tailored to SMSCs has not yet been established. This results in a fragmented understanding of the mechanisms driving HIV/AIDS epidemics in SMSCs, leaving the allocation of prevention and control resources without solid evidential support. Consequently, it is difficult to develop targeted interventions based on the spatiotemporal behaviors of specific populations, ultimately trapping these cities in a predicament where HIV/AIDS prevention and control efforts can only passively keep up with the evolution of the epidemic.

China is the largest developing country in the world. In recent years, the HIV prevalence rate in China has been growing exponentially [7]. In 2009, HIV had already become the main cause of infectious death in China [8]. Data show that the incidence rate of AIDS in China has been steadily increasing over the past 20 years, and the situation is severe [9,10]. Owing to differences in economic development, population density, and meteorological factors, there are spatial differences in the distributions of infectious diseases [11]. The prevalence of AIDS has shifted from a random distribution to spatial clustering. There is obvious spatial heterogeneity among different provinces, and spatial dependence is becoming increasingly strong. As a typical SMSC in China, Nanchang is located in central China and is a core city in the middle and lower reaches of the Yangtze River [12], within its jurisdiction lies Poyang Lake, the largest lake in China [13]. Moreover, there are significant differences in population distribution among the districts and counties of Nanchang, and urban construction and economic development are unbalanced. All these factors make the prevalence characteristics of HIV/AIDS in Nanchang rather complex, suggesting that the prevalence of HIV/AIDS in Nanchang may have corresponding spatiotemporal characteristics.

Considering the above factors, on the basis of the HIV/AIDS surveillance data of Nanchang from 2012–2021, this study innovatively integrates the joinpoint regression model and spatiotemporal geographic information technology to systematically analyze the spatiotemporal evolution patterns of the incidence and mortality of HIV/AIDS as well as the distribution characteristics of the affected population. The study adopts a multidimensional analysis method: joinpoint regression is used to reveal the temporal inflection points of risk evolution, SatScan is applied for spatiotemporal scanning to detect clustered epidemics, and ArcGIS 10.2 software is utilized to conduct spatial autocorrelation analysis (global Moran’s *I*) and cold–hot spot analysis (Getis‒Ord Gi*). For the first time, an epidemiological analysis framework integrating “time–space–population” data for HIV/AIDS patients was constructed in Nanchang. This study bridges a critical gap in the understanding of regional HIV/AIDS transmission patterns, offering robust theoretical support for tailoring interventions to vulnerable populations.

## Methods

### Study area

Nanchang is located in the central north of Jiangxi Province in south-central China (28°09’-29°11’N, 115°27’-116°35’E), with a total area of 7,195 square kilometers and a 2020 resident population of 6.25 million [14]. As the core city of the city cluster in the middle reaches of the Yangtze River, Nanchang is the political, economic, cultural and transportation hub of Jiangxi Province. The city area includes the main urban areas of Donghu District, Xihu District, Qingyunpu District, Qingshan Lake District, Honggutan District and other suburban counties, such as Nanchang County, Anyi County and Jinxian County, with a high topography from southern to northern China, and the Ganjiang River passes through the city, which has a subtropical humid climate with an annual precipitation of 1,600 millimeters, which provides specific ecological and humanistic environments for the transmission of HIV. human environment for HIV transmission.

### Data sources

Data were obtained from the Nanchang Disease Control and Prevention Information System, and cases were screened according to the current address in Nanchang and the date of final examination (1 January 2012–31 December 2021). Demographic information was obtained from the Statistical Yearbook of the Nanchang City Bureau of Statistics. The following variables were collected in this study: age, sex, year of diagnosis and death, and usual residence, and the data were double-checked to eliminate inconsistencies and redundancies.

### Spatiotemporal aggregation scanning analysis

On the basis of the discrete Poisson distribution model, SaTScan 10.2.5 was used to perform spatiotemporal scanning analysis on HIV/AIDS case data from 2012–2021. The scanning analysis was conducted with a maximum radius of 50% of the total population, a time period of 50% of the study cycle, and 999 Monte Carlo simulations. The log likelihood ratio (LLR) statistic was constructed using the actual number of incidence cases to evaluate the degree of abnormality of incidence cases within the scanning window. When the LLR had a P < 0.05 and the relative risk (RR) was significant, the region was considered to have aggregation characteristics. ArcGIS 10.2 was used to visualize the spatiotemporal scanning aggregation regions of the HIV/AIDS cases in Nanchang.

### Spatial autocorrelation analysis

Spatial autocorrelation can analyze the correlation of epidemics at different spatial locations and measure the degree of aggregation of spatial unit attribute values [15]. Spatial autocorrelation was analyzed via ArcGIS 10.2 software to analyze the reported HIV/AIDS cases in Nanchang.

Global autocorrelation analysis can divide the overall spatial distribution into three categories: clustering, dispersion, and randomness. The Moran’s *I* index is used to identify whether there is overall spatial correlation and its degree to determine whether the aggregated elements in the region are statistically significant at the α = 0.05 level. Its value ranges from [−1,1], and the *Z* value is obtained by hypothesis testing of Moran’s I. When Moran’s *I* > 0 and *Z* > 1.96, it indicates positive spatial correlation, and the larger the value is, the stronger the spatial agglomeration. When Moran’s *I* < 0 and *Z* <−1.96, it indicates negative spatial correlation, and the smaller the value is, the greater the spatial dispersion. When Moran’s *I* approaches 0 and Z ranges from −1.96–1.96, it indicates good independence between regions and no spatial correlation [16].

Local spatial autocorrelation analysis was used to identify the spatial correlation of the components in the study area. There are four types of localized spatial clusters in the local indicators of spatial association (LISA) clustering map [17]: “High-high” clustering, also known as “hotspot” areas (high-incidence regions surrounded by high-incidence regions); “Low-low” clustering, also known as “coldspot” areas (low-incidence regions surrounded by low-incidence regions); “High-low” clustering (high-incidence regions surrounded by low-incidence regions); and “Low-high” clustering (low-incidence regions surrounded by high-incidence regions). The first two types indicate that observations in the region are similar to those in the surrounding region. The other two types indicate spatial outliers, meaning that high values are surrounded by neighboring low values or that low values are surrounded by high neighboring values [18].

### Statistical analysis

By adopting descriptive epidemiological methods, this study analyzes the epidemiological characteristics and trends of HIV/AIDS cases in Nanchang city from 2012–2021. The data were organized and statistically analyzed via Excel 2016 with SPSS 25.0 software. Data on demographic characteristics were subjected to the chi-square test. The direct standardization method was used to calculate the standardized incidence rate (SIR) and standardized mortality rate (SMR) of HIV/AIDS at 1/ 100,000 person years, to account for potential biases from population age-structure variations. The Joinpoint Regression Program 5.3.0 was used to explore the turning points and rates of change in HIV/AIDS trends in Nanchang from 2012 to 2021. With calendar year (2012–2021) as the independent variable to model temporal trajectories and detect trend turning points, the dependent variables included annual HIV infection rates, HIV-related SIR, gender-stratified HIV infection rates (male/female), and age-stratified HIV infection rates (0–14, 15–64, and ≥65 years) for gender-specific and age-specific trend analysis. The joinpoint regression model allows estimation of the annual percentage change (APC) and average APC (AAPC) for each component. ArcGIS 10.2 was used for mapping and analysis of spatial global autocorrelation and local autocorrelation, and SaTScan 10.2.5 spatial scanning was used for visual presentation. Statistical significance was defined as *P* < 0.05. For the small number of missing non-core variables, listwise deletion was applied in the analyses.

### Ethics statements

Ethical Approval and consent to participate: This study has obtained ethical approval from the Nanchang City Disease Prevention and Control Center. Project Name: Study on the Role and Mechanism of Defective Functional HDL in HIV-Related Vascular Endothelial Function Damage. Approval Number: 2023 No. 002.

## Results

### Trends in the incidence and mortality of HIV/AIDS

From 2012–2021, the total population of Nanchang increased from 5.278 million to 6.438 million, with an average annual growth rate of 2.1%. A total of 3,789 new HIV/AIDS cases were reported among the permanent residents of Nanchang. The number of new AIDS cases continuously increased from 268 in 2012–455 in 2021, with an average annual growth rate of 5.4%. The incidence rate fluctuated from 5.08/100,000 to 7.07/100,000, peaking in 2019 (7.57/100,000). The number of deaths showed a fluctuating downward trend, decreasing from 91 cases in 2012–52 cases in 2021, and the mortality rate decreased from 1.72/100,000 to 0.81/100,000. Notably, the mortality rate decreased significantly in 2021, with a 40.9% decline compared with that in 2020. Notably, there was an abnormal peak in the number of deaths and mortality rate in 2013 (n = 137, 2.55/100,000), followed by a gradual decrease annually. The changes in both incidence rates and mortality rates were statistically significant (AAPC = 3.85% and −7.76%, t = 4.88 and −4.18, respectively; all *P* < 0.005).

**Fig 1:**
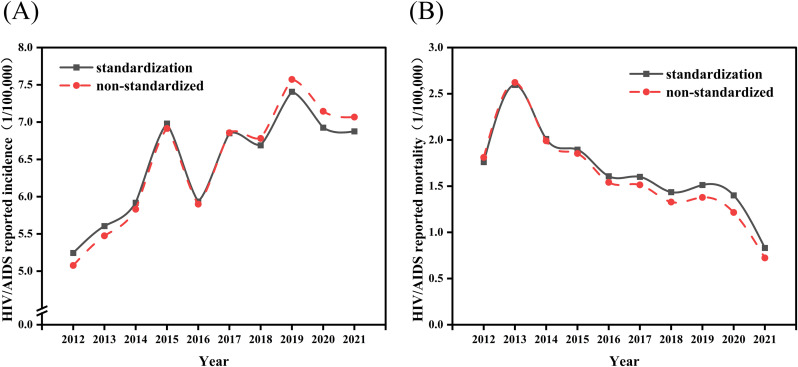
Reported incidence (A) and mortality (B) of HIV/AIDS in Nanchang, 2012‐2021.

Overall, after standardization, the overall incidence and mortality rates of AIDS in Nanchang decreased compared with the unstandardized rates over the 10 years from 2012–2021, dropping from the unstandardized rates of 6.50/100,000 and 1.61/100,000 to 6.47/100,000 and 1.55/100,000, respectively. The standardized incidence and mortality rates are shown in Fig 1.

### Population characteristics

#### Gender- and age-specific trends in HIV/AIDS incidence and mortality.

Gender-specific analysis revealed significantly higher HIV/AIDS incidence and mortality rates among males than females in Nanchang from 2012–2021. Notably, as Table 1 shows, males presented a marked increasing trend in incidence (AAPC = 4.08%, *t* = 4.88, *P* < 0.005). In contrast, mortality rates declined significantly in both genders (AAPC = −7.71% for males, *t* = −4.01; AAPC = −9.23% for females, *t* = −2.71; bo*t*h *P* < 0.05), with females showing a faster decline than males.

**Table 1:** Gender-specific HIV/AIDS incidence and mortality rates in Nanchang city, 2012–2021 (1/100,000).

Year	Incidence		Mortality	
	male	female	male	female
2012	8.24	1.62	2.90	0.44
2013	8.74	1.91	4.28	0.66
2014	9.50	1.83	3.35	0.46
2015	11.57	1.83	3.22	0.37
2016	10.03	1.38	2.66	0.36
2017	10.95	2.37	2.49	0.53
2018	11.66	1.43	2.54	0.17
2019	12.23	2.46	2.46	0.41
2021	12.03	1.78	2.38	0.27
Total	10.72	1.89	2.73	0.38
AAPC (%)	4.08	2.19	−7.71	−9.23
95%CI (%)	(2.13, 6.06)	(−2.77, 7.41)	(−11.87, −3.35)	(−16.42, −1.43)
*t* value	4.88	1.01	−4.01	−2.71
*P* value	0.001	0.344	0.004	0.027

As shown in S3 and S4 Tables, HIV/AIDS incidence and mortality rates in Nanchang city remained near zero among individuals aged <15 years from 2012–2021, with gradual increases observed after age 15 years and significant increases beyond age 65 years. Notably, permanent residents aged 15–64 years presented a substantial upward trend in incidence (AAPC = 4.92%, *t* = 4.04, *P* < 0.05) alongside a significan*t* decline in mortality (AAPC = −10.72%, *t* = −5.31, *P* < 0.05). In contras*t*, those aged ≥65 years showed a nonsignificant downward trend in incidence (AAPC = −8.88%, *t* = −3.69, *P* < 0.05 for mortality) bu*t* demonstrated a statistically significant mortality reduction.

#### Career and contact history in HIV/AIDS incidence.

**Fig 2:**
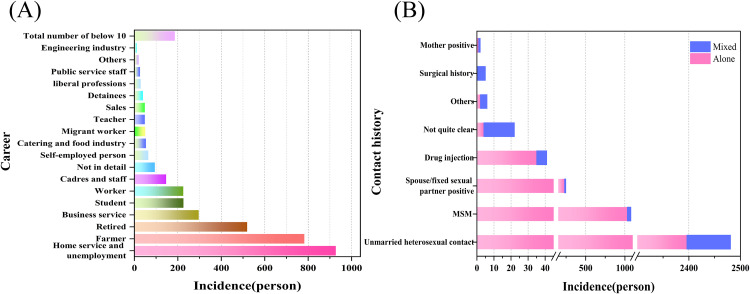
Reported Career (A), Contact history (B) of HIV/AIDS in Nanchang, 2012‐2021.

The occupational distribution of HIV/AIDS cases in Nanchang city from 2012–2021 demonstrated significant clustering (Fig 2A). The three predominant groups were home workers and unemployed individuals (927 cases, 24.47%), farmers (782 cases, 20.64%), and retirees (519 cases, 13.70%), collectively accounting for 58.81% of the total cases. Other notable occupations included business service workers (296 cases, 7.81%), students (226 cases, 5.96%), and workers (225 cases, 5.94%). Among the secondary occupational groups, the proportions of self-employed people (64 cases, 1.69%), those in the catering and food industry (53 cases, 1.40%), and migrant workers (50 cases, 1.32%) were all less than 2%. The number of cases in all other occupations was less than 50, and the occupational information of 94 patients was not clearly recorded. The data indicated that most occupations had fewer than 10 cases, with the occupational distribution of cases predominantly comprising groups with high mobility or weak social support, exhibiting long-tail characteristics.

In terms of the contact history of the affected population, as shown in Fig 2B, the transmission routes of HIV/AIDS patients also demonstrated centralized characteristics. Sexual contact is the core route, with unmarried heterosexual contact being the main transmission pathway. Among them, 65.5% (n = 2,481) of patients had a history of unmarried heterosexual contact, whereas MSM (men who have sex with men) was the second most common route, accounting for 28.4% (n = 1,078), of which 95.5% (n = 1,030) involved a single transmission route. Positive spousal/fixed sexual partner status and injection drug use history accounted for 6.5% (n = 247) and 1.1% (n = 41), respectively. In addition, cross-transmission analysis revealed that 44 patients (1.2%) had both unmarried heterosexual contact and a history of MSM. Special transmission routes accounted for less than 0.2%, including 5 patients (0.13%) with a history of surgery and 2 patients (0.05%) with mother-to-child transmission. The transmission routes of the remaining 22 patients were unknown.

### Spatiotemporal aggregation scanning analysis

From 2012–2021, a total of 3,789 HIV/AIDS cases were reported in Nanchang city. Donghu District presented the highest average incidence rate of AIDS, at 9.99 per 100,000 people, during this decade, whereas Anyi County presented the lowest average incidence rate (4.55 per 100,000 people). The average incidence rate in Donghu District was 2.2 times greater than that in Anyi County. In terms of cumulative case numbers, Nanchang County recorded the highest count (n = 639), followed by Donghu District (n = 466) and Qingshanhu District (n = 433). Anyi County had the lowest cumulative number of cases (n = 104), with Nanchang County reporting 6.14 times more cases than Anyi County did (Table 2).

**Table 2:** The number of reported HIV/AIDS cases and incidence by district, Nanchang, 2012–2021.

District	Cases per district, n/N (per 100,000)										
	2012	2013	2014	2015	2016	2017	2018	2019	2020	2021	Total
Donghu	41/475806(8.62)	42/476273(8.82)	59/499968(11.80)	48/499011(9.62)	42/497998(8.43)	58/475090(12.21)	51/465819(10.95)	50/440899(11.34)	38/421744(9.01)	37/412925(8.96)	466/4665533(9.99)
Xihu	36/514796(6.99)	38/519854(8.71)	35/525112(6.67)	46/529388(8.69)	31/533666(6.00)	47/520317(9.03)	35/516892(6.77)	58/508517(11.41)	64/485223(13.19)	43/482904(8.90)	434/5136669(8.45)
Qingyunpu	16/327613(4.88)	19/332749(5.71)	24/338479(7.09)	31/344669(8.99)	20/344597(5.80)	27/345429(7.82)	29/346076(8.38)	30/348719(8.60)	34/349119(9.74)	29/357410(8.11)	259/3434860(7.54)
Qingshanhu	33/603925(5.46)	42/611017(6.87)	33/603025(5.47)	46/604329(7.61)	42/616467(6.81)	43/635986(6.76)	40/645009(6.20)	51/654351(7.79)	55/662722(8.30)	58/675610(8.58)	443/6312441(7.02)
Xinjian	22/626999(3.51)	26/630496(4.12)	21/634188(3.31)	24/638957(3.76)	35/644151(5.43)	34/653934(5.20)	56/658576(8.50)	48/663965(7.23)	53/676116(7.81)	54/640291(8.43)	373/6467673(5.77)
Honggutan	13/290835(4.47)	19/305651(6.22)	26/322135(8.07)	34/340476(9.99)	35/368056(9.51)	33/411748(8.01)	23/443488(5.19)	29/493436(5.88)	23/555826(4.14)	31/594198(5.22)	266/4125849(6.45)
Nanchangxian	51/894843(5.70)	45/924336(4.87)	53/957297(5.54)	80/991489(8.07)	62/1041378(5.95)	71/1084445(6.55)	56/1121016(5.00)	80/1152431(6.94)	60/1186972(5.05)	81/1227211(6.60)	639/10581418(6.04)
Anyi	4/196487(2.04)	6/201935(2.97)	6/208913(2.87)	11/216215(5.09)	6/226128(2.65)	9/232614(3.87)	18/240234(7.49)	19/244397(7.77)	9/252623(3.56)	16/265336(6.03)	104/2284882(4.55)
Jinxian	21678813(3.09)	25/675884(3.70)	30/672973(4.46)	29/670185(4.33)	22/667186(3.30)	32/664527(4.82)	40/660958(6.05)	31/642826(4.82)	29/620334(4.67)	35/643481(5.44)	294/6597167(4.46)
Jinkai	14/324363(4.32)	11/337324(3.26)	14/353489(3.96)	15/370710(4.05)	21/392582(5.35)	24/412120(5.82)	22/423027(5.20)	31/465565(6.66)	32/486649(6.58)	30/561415(5.34)	214/4127244(5.19)
Gaoxin	17/260968(6.51)	21/268641(7.82)	19/280445(6.77)	23/293329(7.84)	22/312381(7.04)	28/381167(7.35)	38/386165(9.84)	38/415291(9.15)	50/447178(11.18)	41/463175(8.85)	297/3508740(8.54)

**Fig 3:**
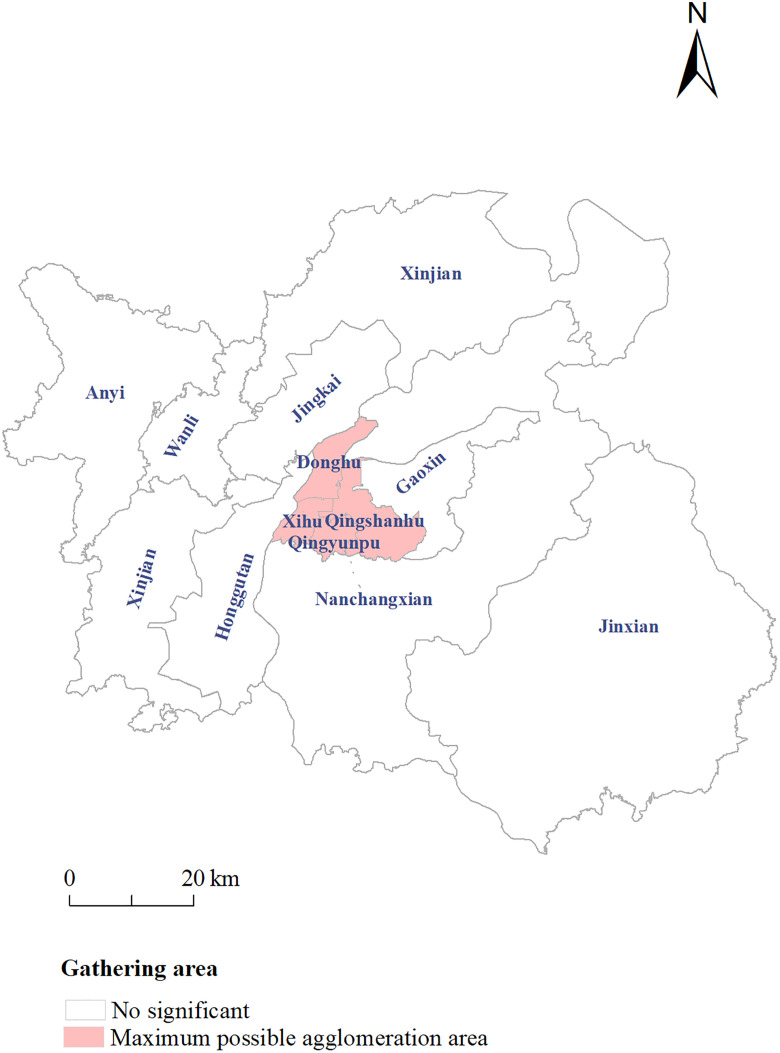
Spatiotemporal scan analysis of the number of reported HIV/AIDS cases in Nanchang.

A statistically significant disease cluster, encompassing the Xihu, Donghu, Qingshanhu, and Qingyunpu districts, was identified in Nanchang city between 2012 and 2021. The cluster exhibited a temporal span of 2017–2021, with a spatial radius of 11.95 km (RR = 1.47, LLR = 46.10, *P* < 0.01). The geographical distribution of this high-risk cluster is shown in Fig 3.

### Spatial autocorrelation analysis

#### Global spatial autocorrelation analysis.

From 2012–2021, HIV/AIDS cases in Nanchang city exhibited spatial clustering (Moran’s I = 0.1403, Z = 1.9839, *P* = 0.04726). Annual analyses via the global Moran’s I index revealed values of 0.1779 (Z = 2.145803, *P* = 0.031889) in 2012, 0.1971 (Z = 2.273913, *P* = 0.022971) in 2013, 0.1604 (Z = 2.039396, P = 0.041411) in 2016, and 0.189 (Z = 2.187793, *P* = 0.028685) in 2020, indicating spatial clustering and positive spatial correlation in these years. The Moran’s I values for the remaining years were not statistically significant, suggesting that there was no spatial correlation or clustering in the reported incidence of HIV/AIDS in Nanchang city during these periods (Table 3).

**Table 3:** Overall spatial autocorrelation analysis of HIV/AIDS cases in Nanchang city from 2012–2021.

Year	Moran’s *I*	*Z* value	*P* value	Distribution type
2012	0.178	2.146	0.032	Clustered
2013	0.197	2.274	0.023	Clustered
2014	0.114	1.684	0.092	Random
2015	0.144	1.850	0.064	Random
2016	0.160	2.039	0.041	Clustered
2017	0.130	1.858	0.063	Random
2018	−0.117	−0.222	0.825	Random
2019	−0.045	0.397	0.691	Random
2020	0.189	2.188	0.029	Clustered
2021	0.065	1.406	0.160	Random
Average	0.140	1.984	0.047	Clustered

#### Local spatial autocorrelation analysis.

**Fig 4:**
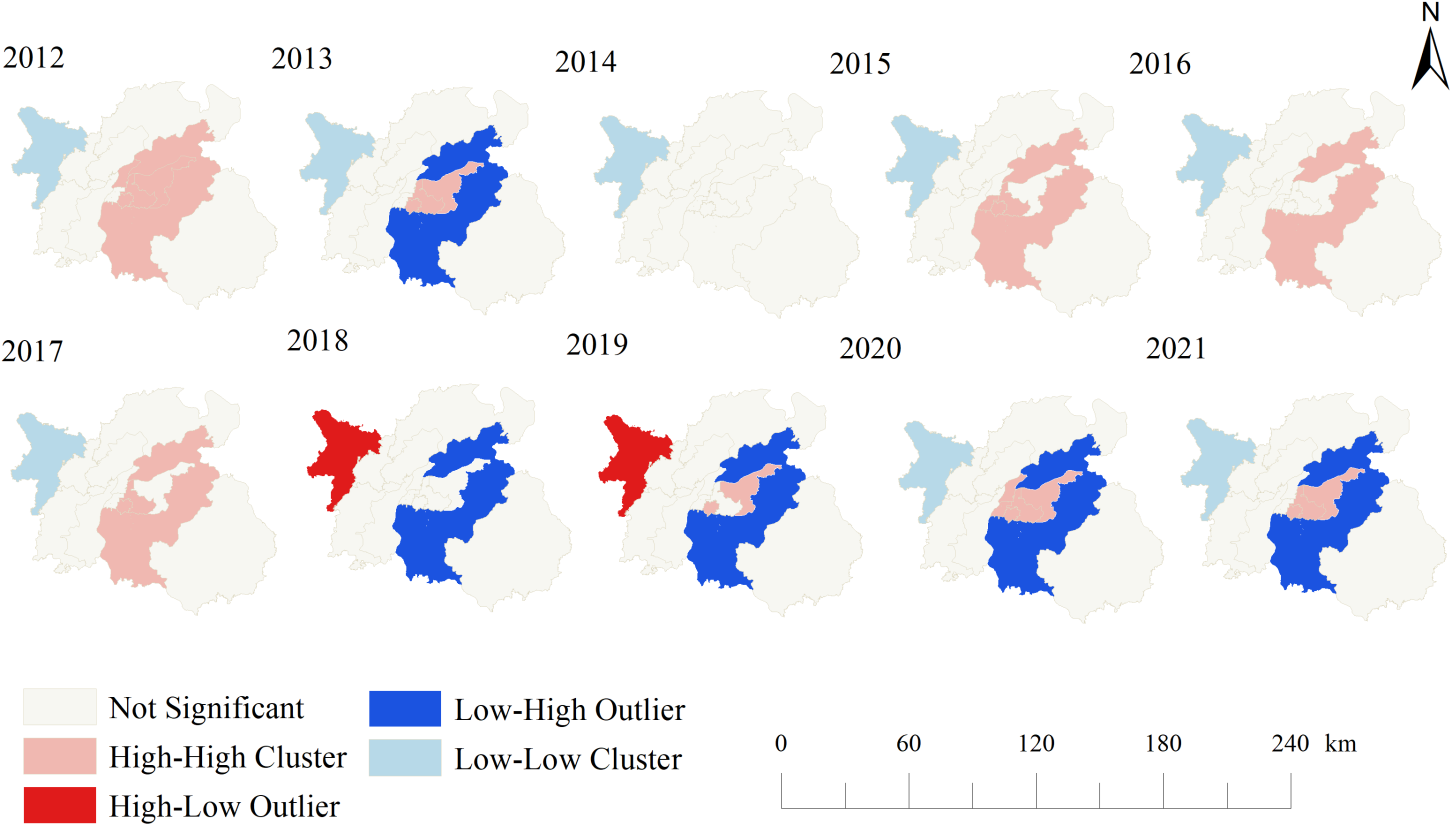
Yearly LISA cluster maps for the reported incidence of HIV/AIDS, Nanchang, 2012‐2021. Four different colors represent 4 types of local spatial clustering: high-high cluster, low-low cluster, low-high outlier, and high-low outlier.

As shown in Fig 4, local spatial autocorrelation analysis of HIV/AIDS cases in Nanchang city from 2012–2021 revealed four types of clustering patterns—“high-high,” “high-low,” “low-low,” and “low-high”—in the LISA cluster map over this decade. The spatial distribution of HIV/AIDS cases in Nanchang city from 2012–2021 exhibited significant regional differentiation. Hotspot areas were primarily concentrated in the central urban area and adjacent regions. In 2012, the hotspot coverage was the widest, including Nanchang County, the high-tech industrial development zone, Donghu District, Qingshanhu District, and Qingyunpu District. From 2013–2017, hotspots gradually contracted toward Xihu District, Qingshanhu District, and Qingyunpu District. In 2016, the hotspot area was the smallest, with only Nanchang County showing continuous clustering. In 2020, hotspots expanded again to cover the high-tech industrial development zones of Xihu District, Donghu District, Qingshanhu District, and Qingyunpu District, forming a contiguous distribution. By 2021, the hotspot area had retracted to the junction of the high-tech industrial development zone, Qingshanhu District, and Qingyunpu District.

The cold spot remained stable in Anyi County throughout the study period, with only brief abnormal fluctuations in its northwestern region from 2018–2019. Additionally, Nanchang County exhibited a unique “hot‒cold alternation” pattern: from 2013 and 2018–2021, the area shifted from a hotspot to an outlier pattern where low values were surrounded by high values, whereas central urban areas such as Xihu District and the high-tech industrial development zone remained consistently hotspots or in transitional states. During the study period, Qingshanhu District, Qingyunpu District, and the high-tech industrial development zone served as core hotspot anchors, whereas Anyi County, as a coldspot area, formed a striking spatial polarization pattern with hotspots.

## Discussion

This study revealed that from 2012–2021, there was a significant “scissor gap” characteristic between the number of new HIV/AIDS cases and population growth in Nanchang: the average annual population growth rate (2.1%) was much lower than the case growth rate (5.4%), indicating that the accelerating trend of HIV transmission may be associated with multiple factors, including increased engagement in high-risk behaviors, expanded testing coverage, and the clustering of mobile populations. For instance, Nanchang launched HIV proactive testing in medical institutions at or above the county level in 2012, which not only broadened the scope of testing but also enhanced the case detection rate. By 2018, the city had further rolled out a policy to realize full coverage of HIV screening for pregnant women and parturients. Although the incidence rate has continued to rise, the mortality rate has decreased significantly. This may be due to the implementation of the free ART program in China in 2003 and the adoption of the “treatment for all” policy in 2016 [19], which popularized antiretroviral therapy (ART), extended the survival period of patients, and optimized early intervention strategies. The abnormal peak in the mortality rate in 2013 may be related to the lag in early case diagnosis or insufficient treatment compliance. The sharp decline in both the incidence and mortality rates from 2020–2021 may be associated with home isolation and reduced mobility caused by the global COVID-19 pandemic.

This study revealed that, from 2012–2021, there were significant sex and age differences in the HIV/AIDS epidemic in Nanchang. The incidence rate of HIV among men continues to rise and is significantly higher than that among women, which may be attributed to two factors: first, men have a higher exposure rate to high-risk behaviors, such as male-to-male sexual contact and commercial sexual activities; second, men show relatively low willingness and initiative to participate in voluntary HIV testing. This trend is consistent with the overall increase in HIV infection risk among men nationwide [20]. In terms of underlying causes, some men may face greater social pressure, which makes them more vulnerable to engaging in high-risk sexual behaviors. Meanwhile, the social and cultural perception that “men should take the initiative in initiating sexual activities” may lead them to neglect safety precautions during sexual encounters. Additionally, men generally pay less attention to their own health compared with women, with insufficient knowledge of HIV prevention and weak awareness of active testing. In some countries and regions, the coverage of HIV prevention education and promotional activities targeting men is inadequate, which further exacerbates this issue. Notably, the proportion of men in intravenous drug user populations is relatively higher, which also reflects, from another perspective, that men have a higher participation rate in high-risk behaviors [21]. Notably, the incidence rate among the elderly population (≥65 years old) was significantly greater than that among the other groups, and the decline was not obvious. The high incidence rate highlights the lack of sexual health education for the elderly population and the shortcomings of prevention and control, which mainly rely on passive testing. This may be because the overall educational level of elderly individuals over 60 years old in China is low, and their understanding of their sexual life and AIDS is insufficient. Many elderly men still have high enthusiasm for their sexual life, and neglect and indifference from their families often lead to the occurrence of prostitution among elderly people, which provides a way for the transmission of AIDS [22]. Studies have shown that people aged 50 and above are more likely to report risks of heterosexual transmission. This is because, although the rate of new HIV infections among the elderly remains stable, younger individuals who were infected with HIV in earlier years are surviving and aging, which has exacerbated the continuous growth in the number of elderly HIV-infected individuals [23]. Some elderly cases reside in urban-rural fringe areas, where there is a relative scarcity of geriatric clinics providing HIV diagnosis and treatment services. Additionally, factors such as older age, male gender, being single/divorced, and being a migrant population are all associated with delayed diagnosis [24]. However, the significant decrease in the mortality rate among the elderly population indicates that the increased coverage of antiviral treatment has improved the prognosis. The significant increase in the incidence rate (AAPC = 4.92%) and the rapid decrease in the mortality rate (AAPC = −10.72%) among the working-age population aged 15–64 years sharply contrast, which may reflect the dual effects of increased high-risk behaviors and improved treatment accessibility among young and middle-aged groups. In conclusion, it is urgent to carry out precise behavioral interventions for men and elderly individuals and to break the vicious cycle of “late detection - high mortality” by strengthening proactive screening.

In terms of occupational distribution, HIV infections are highly concentrated in three major groups: home workers/unemployed individuals, farmers, and retired personnel, which may be because unstable economic incomes may lead to high-risk behaviors (such as unprotected commercial sex and temporary sexual partnerships) or limited access to medical resources; retired personnel may meet their needs through commercial sexual services due to social isolation or increased spousal mobility, which corroborates the high proportion (65.5%) of unmarried heterosexual contact history; the infection risks of commercial service workers and students may be related to their occupational mobility or sexual openness; and the hidden transmission of MSM among young students needs to be vigilantly monitored. In addition, the long-tail feature of occupational distribution indicates that beyond high-risk groups, the HIV transmission network may penetrate broader social structures through sporadic occupational intersections such as those involving individual practitioners and migrant workers. Studies have shown that individuals with lower education levels, farmers, and those who are divorced or widowed are more likely to be infected with HIV [25]. Future prevention and control should focus on targeted interventions and cross-occupational joint prevention: strengthening sexual safety education and regular screening for vulnerable groups such as home workers and farmers by combining their contact history characteristics; guarding against cross-transmission in highly mobile groups such as business service workers and students; and establishing a multidepartmental collaborative postexposure prophylaxis service system.

Unsafe sexual behavior has been identified as the main cause of HIV infection [26]. Unmarried heterosexual contact history and MSM history constitute the main exposure routes in the study population, a distribution characteristic highly consistent with the trend that sexual transmission has accounted for more than 96% of HIV infections in China in recent years [20,27]. The proportion of unmarried heterosexual contact history as high as 65.48% may reflect social and behavioral risks such as active sexual behavior among mobile populations and unprotected commercial sexual activities, suggesting the need to strengthen safe sex interventions in key labor export regions. The group with a history of male-to-male sexual behavior accounts for 28.45%, which is close to the 26% reported in China’s overall MSM population [28], and 44 of them are at high risk of having unmarried heterosexual contact. For years, the prevalence of HIV infection in MSM has been increasing, with the internet being the main venue for Chinese MSM to seek male sexual partners [25]. This group is characterized by strong concealment and significant tendencies toward multiple sexual partners. It is recommended that HIV self-testing and preexposure prophylaxis (PrEP) services be pushed precisely through social media [29]. Patients with a history of mixed contact accounted for 2.43% (92/3789) of all patients, especially those with the “unmarried heterosexual + MSM” dual-exposure pattern, highlighting that complex behavioral networks may accelerate virus transmission. The bridging role of such populations requires vigilance, and special reports on combined contact history types should be added to sentinel surveillance. The strengthening of interventions in commercial sexual activity gathering places and the development of precise prevention and control strategies for cross-risk behavior groups are recommended.

The spatiotemporal scanning analysis revealed that the spatial distributions of the cases presented significant heterogeneity. As shown in Fig 3, the main spatial clustering area is located in the central urban area of Nanchang, namely, Donghu District, Xihu District, Qingshanhu District and Qingyunpu District. This may be because this area has the highest risk of HIV infection during the study period, which is related to factors such as high population density; the concentration of transportation hubs such as Nanchang Railway Station, Hongcheng Grand Market; and the large floating population. In addition, there are relatively more medical and health institutions in urban areas with abundant medical resources and many patients seeking medical treatment, making it easier to detect cases. Most of the hospitals designated for AIDS in Nanchang are located in this clustered area. For example, as the medical and health center of Nanchang city and even Jiangxi Province, Donghu District has a total of 208 medical institutions of various types, among which there are as many as 12 Class-A tertiary hospitals [30]. Timely and accessible HIV testing is helpful for identifying more HIV/AIDS cases, which is very important for the detection of HIV-infected individuals [31]. Existing studies generally suggest that the spatial characteristics of sexual networks are among the core drivers of HIV geographic clustering [32], and this association is particularly prominent in urban areas, closely aligning with the spatial concentration of MSM and unsafe sexual behaviors. On one hand, LGBTQ + -themed bars, social venues, and other spaces in urban core areas, along with the MSM population attracted by the inclusive environment, jointly form localized sexual network, which create conditions for viral transmission. On the other hand, factors such as weak social support among urban mobile populations and the fast-paced, anonymous social culture in cities have led to a higher incidence of unsafe sexual behaviors in urban areas. The clustering area highly coincides with the main roads in the old urban area and the routes of subway lines, which also implies that transportation corridors may indirectly increase the transmission risk by promoting population mobility. Moreover, the most likely clustering area overlaps spatially with the urban commercial center and the concentrated area of universities. As a transitional zone, the urban-rural fringe sees agricultural populations commute daily to construction sites or informal markets in the city, serving as a bridge connecting rural livelihoods to the high-risk environment of urban areas. Elderly populations, in turn, mainly reside in these urban-rural fringe areas. These findings suggest that attention should be paid to the potential driving role of high-density social activities in the transmission network.

The analysis of global autocorrelation and local spatial autocorrelation reveals the dynamic correlations and driving characteristics of the spatial distribution of HIV/AIDS cases in Nanchang from 2012–2021. First, the global Moran’s *I* index shows that the overall cases exhibit weak positive spatial correlation, but there is significant annual heterogeneity. The Moran’s *I* values in years with high clustering (2012, 2013, 2016, and 2020) all exceed 0.15 (*P* < 0.05), which is consistent with the spatial expansion of local areas with high-high clustering. For example, in 2013, high-high clustering covered the high-tech Zone, Qingshanhu District, and Qingyunpu District, and in 2020, the high-high clustering areas were still the high-tech Zone, Qingshanhu District, and Qingyunpu District, all of which are in the central urban area. The population density of Nanchang increased from 2012–2021, corresponding to a period of rapid urban expansion, with a sharp increase in the floating population and an increased risk of AIDS infection. The local analysis further reveals the evolution mechanism of the “core-periphery” polarization pattern. Central urban areas such as Qingshanhu District and Qingyunpu District serve as the core of persistent high-high clustering, which is related to population density and the density of commercial and entertainment venues, suggesting the driving effect of dense social networks on transmission. Anyi County, as a long-term low-low clustering area, briefly became a high-low outlier from 2018–2019, which may be related to fluctuations in the number of imported cases caused by migrant workers returning home across regions. Notably, Nanchang County shows significant characteristics of cold–hot spot conversion: low–high outliers (low-incidence areas surrounded by high-incidence areas) occurred in 2013 and from 2018–2021. This may be due to its proximity to the main urban area and being affected by the main urban area, indicating that the focus of prevention and control needs to shift toward the “diffusion buffer zone”. Other outer districts and counties did not show statistically significant case clustering in most years. These areas are far from the central urban area, are composed mainly of agricultural populations, and have low spatial mobility, which may be weakly related to the risk of disease transmission. Studies have shown that the spatial association between HIV prevalence and poverty levels varies with the degree of urbanization [33], and this association is particularly pronounced in cities with frequent population mobility. During periods of rapid urban expansion, uneven resource distribution tends to make specific areas—such as urban-rural fringe zones with concentrated migrant populations and downtown areas with dense commercial activities—vulnerable to becoming high-risk carriers for disease transmission. Additionally, HIV cases are not randomly distributed but often cluster in specific geographic units, forming well-defined high-risk agglomerations [34,35], a pattern also observed in the epidemiological distribution of Nanchang from 2012 to 2021.Specifically, the continuous expansion of Nanchang’s metro lines and intercity bus networks between 2017 and 2021 significantly shortened commute times between central urban areas and suburban agglomerations, directly breaking down geographic barriers to population mobility. The marked increase in cross-regional mobility further facilitated the spread of the virus from high-risk clusters to surrounding areas, acting as a critical transmission link connecting core high-risk zones and peripheral potential risk zones.

### Limitations

The limitations of this study include that data are derived from a passive surveillance system, without accounting for underreporting, misreporting, variations in testing capacity, and the status of HIV surveillance among key populations. This may lead to discrepancies between the reported incidence and the actual incidence. Additionally, the study is based on surveillance data rather than behavioral survey data, making it impossible to directly analyze the impact of individual behaviors on infection risk. Furthermore, the depth of analysis is insufficient. The study primarily focuses on qualitative descriptions of factors such as population density, transportation, and medical resources, while the analysis of the driving factors behind the spatial distribution of HIV is relatively superficial. It fails to quantitatively analyze the contribution of each factor and spatial heterogeneity. Meanwhile, the conclusions of this study are only applicable to Nanchang City, and their generalizability to other regions is subject to certain limitations.

This study did not fully capture potential confounding factors such as income level and educational attainment. These variables may be correlated with both occupational characteristics and HIV infection risk, which could affect the precision of some analysis results. Future studies could integrate more detailed socioeconomic data to further clarify the complex associations among the above variables and enhance the robustness of conclusions.

## Conclusions

In conclusion, on the basis of the HIV/AIDS case data of Nanchang city from 2012–2021, this study innovatively integrates the joinpoint regression model and spatiotemporal geographic information technology, systematically revealing the spatiotemporal evolution patterns of the incidence and mortality of the disease, the distribution characteristics of the population, and its potential influencing factors. The study confirmed that the growth rate of new HIV/AIDS cases was significantly greater than the population growth rate during the same period and that there was an inverse relationship between the incidence rate and the mortality rate. This phenomenon is closely related to increased exposure to high-risk behaviors, improvements in testing coverage, and the widespread implementation of antiviral treatment policies. In terms of prevention and control strategies, targeted health education and publicity interventions should be carried out according to the differentiated infection risks and prevention and control difficulties faced by male and elderly populations. Moreover, eliminating the sexual transmission route should be regarded as the core focus of AIDS prevention and control work, and the prevention and control of sexual transmission risk should be strengthened. The results of spatial analysis show that the central urban area of Nanchang city is the main clustered area of cases, and its clustering trend is significantly related to population density, the layout of transportation hubs, the intensity of commercial activities, and the distribution of universities. In light of this, in central urban areas with “high-high” clustering, HIV/AIDS health education should be further strengthened to improve public awareness, and testing coverage should be expanded to achieve the early detection of infected individuals. For areas with “low-low” clustering, effective epidemic monitoring and prevention and control barriers need to be established to prevent the spread of the epidemic from surrounding high-risk areas.

Based on the research results, strategies can be implemented from two aspects: urban management and precision prevention and control. From the urban management perspective, AIDS prevention and control knowledge should be incorporated into “citizen health literacy”, and awareness should be improved through “five-entry” publicity (into communities, enterprises, hospitals, schools, and families) and thematic activities on key occasions. Meanwhile, condom vending machines should be added in areas with concentrated floating populations and universities to ensure full coverage of condoms in hotels and other public places, and an intelligent monitoring and early warning system should be built relying on big data. From the precision prevention and control perspective, popular lectures should be held for the elderly, new media information should be pushed for men who have sex with men, and multilingual science popularization should be provided for floating populations. In addition, internet can be used to realize the linkage between online risk assessment and offline testing, and friendly health centers should be set up in core areas to provide anonymous testing and psychological support.

From the perspective of global urban applicability, universal knowledge popularization, condom promotion, basic internet, and intelligent monitoring are universal measures. Small and medium-sized cities in developing countries can use these to quickly improve their prevention and control capabilities, while large cities in developed countries can integrate them into local systems, and cities with weak informatization can also promote them in a simplified way. Small and medium-sized cities should focus on “low-cost and wide-coverage” community publicity and mobile testing.

This study systematically illustrates the intricate characteristics of the HIV/AIDS epidemic in Nanchang city. By revealing spatiotemporal patterns and population-specific risk factors, this research offers robust theoretical underpinnings and practical frameworks for tailoring targeted interventions for vulnerable groups, formulating evidence-based medical resource allocation strategies, and strengthening interdepartmental cooperation in prevention and control efforts. These contributions are instrumental in enhancing the effectiveness of public health interventions, reducing the transmission rate of HIV/AIDS, and ultimately alleviating the disease burden across the population.

## Supporting information

S1 Table Overall incidence and death of HIV/AIDS in Nanchang. (DOCX)

S2 Table The population characteristics in Nanchang. (DOCX)

S3 Table Age-specific incidence rate of AIDS in Nanchang. (DOCX)

S4 Table Age-specific mortality rate of AIDS in Nanchang. (DOCX)

S1 Data This file contains data on gender, occupation, marital status, and contact history of HIV/AIDS patients in Nanchang from 2012 to 2021. All personally identifiable information has been removed, complying with ethical guidelines and participant consent requirements.
(XLSX)
