# Supplementary material for: Spatiotemporal aggregation and population distribution characteristics of HIV/AIDS in Nanchang city: A monitoring analysis from 2012–2021
Source: PLoS One. 2026 Feb 5;21(2):e0342375. doi: 10.1371/journal.pone.0342375 (PMC12875437; doi:10.1371/journal.pone.0342375)
Supplement: S1 Table — (DOCX) [file pone.0342375.s001.docx]

**Table S1.** Overall incidence and death of HIV/AIDS in Nanchang

| Year | Total population | Incidence  (n) | incidence（1/100,000） | Deaths  (n) | mortality（1/100,000） |
| --- | --- | --- | --- | --- | --- |
| 2012 | 5278077 | 268 | 5.08 | 91 | 1.72 |
| 2013 | 5369614 | 294 | 5.48 | 137 | 2.55 |
| 2014 | 5487421 | 320 | 5.83 | 108 | 1.97 |
| 2015 | 5596603 | 387 | 6.91 | 104 | 1.86 |
| 2016 | 5746168 | 339 | 5.90 | 90 | 1.57 |
| 2017 | 5920796 | 406 | 6.86 | 92 | 1.55 |
| 2018 | 6016213 | 408 | 6.78 | 85 | 1.41 |
| 2019 | 6140465 | 465 | 7.57 | 91 | 1.48 |
| 2020 | 6255814 | 447 | 7.15 | 86 | 1.37 |
| 2021 | 6437506 | 455 | 7.07 | 52 | 0.81 |
