# Supplementary material for: Spatiotemporal aggregation and population distribution characteristics of HIV/AIDS in Nanchang city: A monitoring analysis from 2012–2021
Source: PLoS One. 2026 Feb 5;21(2):e0342375. doi: 10.1371/journal.pone.0342375 (PMC12875437; doi:10.1371/journal.pone.0342375)
Supplement: S2 Table — (DOCX) [file pone.0342375.s002.docx]

**Table S2.** The population characteristics in Nanchang

| Year | Total population | Age (years old) | | | Gender | |
| --- | --- | --- | --- | --- | --- | --- |
|  |  | 0-14 | 15-64 | 65+ | Man | Woman |
| 2012 | 5278077 | 963997 | 3878507 | 435574 | 2754576 | 2523502 |
| 2013 | 5369614 | 968674 | 3940460 | 460480 | 2801702 | 2567913 |
| 2014 | 5487421 | 990220 | 4013709 | 483492 | 2862208 | 2625213 |
| 2015 | 5596603 | 1004009 | 4087643 | 504951 | 2922278 | 2674325 |
| 2016 | 5746168 | 1028232 | 4182314 | 535622 | 3002060 | 2744108 |
| 2017 | 5920796 | 1060076 | 4295304 | 565416 | 3095249 | 2825547 |
| 2018 | 6016213 | 1065949 | 4356368 | 593895 | 3146292 | 2869921 |
| 2019 | 6140465 | 1077089 | 4434852 | 628524 | 3213332 | 2927132 |
| 2020 | 6255814 | 1091014 | 4505377 | 659423 | 3274726 | 2981088 |
| 2021 | 6437506 | 1051376 | 4697208 | 688922 | 3371767 | 3065739 |
