# Supplementary material for: Spatiotemporal aggregation and population distribution characteristics of HIV/AIDS in Nanchang city: A monitoring analysis from 2012–2021
Source: PLoS One. 2026 Feb 5;21(2):e0342375. doi: 10.1371/journal.pone.0342375 (PMC12875437; doi:10.1371/journal.pone.0342375)
Supplement: S3 Table — (DOCX) [file pone.0342375.s003.docx]

**Table S3.** Age-specific incidence rate of AIDS in Nanchang. (1/ 100,000)

| Year | Age specific incidence Age (years old) | | | Total incidence | |
| --- | --- | --- | --- | --- | --- |
|  | 0-14 | 15-64 | 65+ | non-standardized | standardized |
| 2012 | 0 | 4.85 | 18.37 | 5.08 | 5.24 |
| 2013 | 0.10 | 4.19 | 27.8 | 5.48 | 5.61 |
| 2014 | 0 | 5.48 | 20.68 | 5.83 | 5.92 |
| 2015 | 0 | 6.75 | 21.98 | 6.91 | 6.98 |
| 2016 | 0 | 6.03 | 16.24 | 5.90 | 5.93 |
| 2017 | 0 | 6.75 | 20.52 | 6.86 | 6.85 |
| 2018 | 0 | 6.29 | 22.56 | 6.78 | 6.69 |
| 2019 | 0 | 7.08 | 24.02 | 7.57 | 7.41 |
| 2020 | 0 | 6.55 | 23.05 | 7.15 | 6.93 |
| 2021 | 0 | 7.24 | 16.69 | 7.07 | 6.88 |
| total | 0.01 | 6.17 | 21.13 | 6.50 | 6.47 |
| AAPC(%) |  | 4.92 | -0.67 | 3.85 | 3.11 |
| 95%CI(%) |  | (2.08, 7.83) | (-5, 3.85) | (2.01,5.73) | (1.31, 4.94) |
| *T* value |  | 4.04 | -0.35 | 4.88 | 4.02 |
| *P* value |  | 0.004 | 0.737 | 0.001 | 0.004 |
