# Supplementary material for: Spatiotemporal aggregation and population distribution characteristics of HIV/AIDS in Nanchang city: A monitoring analysis from 2012–2021
Source: PLoS One. 2026 Feb 5;21(2):e0342375. doi: 10.1371/journal.pone.0342375 (PMC12875437; doi:10.1371/journal.pone.0342375)
Supplement: S4 Table — (DOCX) [file pone.0342375.s004.docx]

**Table S4.** Age-specific mortality rate of AIDS in Nanchang City. (1/100,000)

| Year | Age specific mortality Age (years old) | | | | Total mortality | |
| --- | --- | --- | --- | --- | --- | --- |
|  | 0-14 | 15-64 | | 65+ | non-standardized | standardized |
| 2012 | 0 | 1.01 | | 11.94 | 1.76 | 1.81 |
| 2013 | 0 | 1.27 | | 18.89 | 2.60 | 2.62 |
| 2014 | 0 | 1.12 | | 13.03 | 2.01 | 1.99 |
| 2015 | 0 | 0.98 | | 12.67 | 1.90 | 1.85 |
| 2016 | 0 | 0.98 | | 9.15 | 1.61 | 1.54 |
| 2017 | 0 | 0.98 | | 8.84 | 1.60 | 1.51 |
| 2018 | 0 | 0.64 | | 9.60 | 1.44 | 1.33 |
| 2019 | 0 | 0.77 | | 9.07 | 1.51 | 1.38 |
| 2020 | 0 | 0.44 | | 10.01 | 1.40 | 1.22 |
| 2021 | 0 | 0.40 | | 4.79 | 0.83 | 0.72 |
| Total | 0 | 0.84 | | 10.40 | 1.61 | 1.55 |
| AAPC(%) |  | -10.72 | | -8.88 | -7.76 | -9.52 |
| 95%CI(%) |  | (-15.01, -6.22) | | (-14.03, -3.43) | (-11.79, -3.56) | (-13.45, -5.41) |
| *T* value |  | | -5.31 | -3.69 | -4.18 | -5.19 |
| *P* value |  | | 0.001 | 0.006 | 0.003 | 0.001 |
